# Role of High-Dose Chemotherapy and Autologous Hematopoietic Cell Transplantation for Children and Young Adults with Relapsed Ewing's Sarcoma: A Systematic Review

**DOI:** 10.1155/2018/2640674

**Published:** 2018-06-03

**Authors:** Pavan Tenneti, Umar Zahid, Ahmad Iftikhar, Seongseok Yun, Atif Sohail, Zabih Warraich, Faiz Anwer

**Affiliations:** ^1^Department of Medicine, University of Arizona, Tucson, AZ 85721, USA; ^2^Department of Biostatics, Mel and Enid Zuckerman College of Public Health, University of Arizona, Tucson, AZ 85721, USA; ^3^Division of Hematology and Oncology, Department of Medicine, University of Arizona, Tucson, AZ 85721, USA; ^4^Malignant Hematology, H. Lee Moffitt Cancer Center and Research Institute, Tampa, FL 33612, USA

## Abstract

**Background:**

Relapsed Ewing's sarcoma (RES) is an aggressive malignancy with poor survival. Although high-dose chemotherapy (HDCT) with autologous stem cell transplantation (ASCT) given after conventional chemotherapy (CC) has shown survival benefits, it is not generally used in the United States for RES. We performed a systemic review to evaluate the benefits of HDCT for RES.

**Methods:**

Literature search involved Medline, Embase, and Cochrane database. We included studies with RES patients treated with HDCT/ASCT.

**Results:**

Twenty-four studies with total of 345 reported RES patients that got HDCT were included in final analysis. Seventeen studies had patients with multiple malignancies including RES, while seven had only RES patients. At 2 and 3–5 years, event-free survival (EFS) in studies with only RES patients ranged 42–47% and 20–61% and overall survival (OS) ranged 50–66% and 33–77%, respectively. In studies with combined patients that reported outcomes of RES separately, the EFS at 1–3 and 4 years was 36–66% and 17–50%, respectively. The OS at 1-2 and 3-4 years was 40–60% and 50–70%.

**Conclusions:**

Most studies using HDCT/ASCT as consolidation regimen showed improved survival benefits compared to CC. Randomized controlled studies are needed to determine true clinical benefits of HDCT followed by ASCT in patients with RES.

## 1. Introduction

Patients with localized primary Ewing's sarcoma (ES) have 60–70% 5-year overall survival (OS) with multimodality treatment [1]. In patients with primary metastatic Ewing's sarcoma, the OS rate is 20–40% with treatment [1]. Approximately 30–40% of patients with primary Ewing's sarcoma who initially achieved remission after front-line treatment experience disease relapse, and the prognosis in these patients was shown to be dismal [2]. The poor prognostic factors at relapse include relapse time less than 2 years from initial diagnosis, location of relapse at an extrapulmonary site, combined local as well as systemic relapse, and high lactate dehydrogenase (LDH) levels at initial diagnosis [3–5]. No standardized treatment has been approved for relapsed ES. Local therapy at the site of relapse including radical surgery has shown to be beneficial [5]. Conventional salvage chemotherapy (CC) regimens given at relapse have led to response rates up to 29–68.1% depending on the type of regimen used and site of relapse [6–10]. The event-free survival (EFS) at 10.3 months–2 years is noted to be 22.7–26% in the literature [8, 9]. OS rate at 1-2 years was shown to be about 28–61% [7, 8]. The five-year OS was reported 20–24.5% in a retrospective study [11]. Despite its reported survival benefit, high-dose chemotherapy (HDCT) and autologous stem cell transplant (ASCT) is not routinely used in the United States for relapsed Ewing's sarcoma. In the last fifteen years, there have been two systematic reviews that were performed to evaluate the benefit of HDCT in Ewing's sarcoma, but these studies mainly focused on locally advanced and primary metastatic disease without major focus on relapsed Ewing's sarcoma [12, 13]. We performed a comprehensive literature search and performed systematic review to evaluate the role of HDCT along with ASCT given as an induction or consolidation regimen in patients with relapsed Ewing's sarcoma.

## 2. Materials and Methods

### 2.1. Source of Information and Search Strategy

Comprehensive literature search was conducted using Medline (PubMed and Ovid SP), Embase, and Cochrane Database of Systematic Review (CDSR) up to April 2017. The search strategies included various combinations of text words and controlled vocabulary when available. We used the following keywords while building search; Ewing sarcoma, relapsed/refractory, chemotherapy, high-dose chemotherapy, transplant, tandem transplant, treatment. There was no language or year limits placed on the search. Three reviewers (Pavan Tenneti, Umar Zahid, and Ahmad Iftikhar) independently applied the inclusion and exclusion criteria to the articles that were identified by the search strategy and extracted data using the standardized data extraction form. In addition, more articles were added from bibliographical data of searched articles found through search engines. Full articles of potentially useful articles were reviewed before confirming the inclusion criteria.

### 2.2. Inclusion and Exclusion Criteria

We included all clinical studies that had patients with relapsed Ewing's sarcoma who were treated with HDCT followed by ASCT using either bone marrow or peripheral blood stem cell rescue regardless of treatment settings. We excluded studies that had patients with only primary local or metastatic Ewing's sarcoma or other soft tissue sarcomas and without relapsed disease. As our article mainly aims to compare long-term outcomes of patients that got HDCT to those that did not, we also excluded ten case reports and case series that did not mention long-term outcomes of relapsed ES in their studies. Following data were extracted from the individual study: demographics, pathology, disease stage, treatment, response, and survival outcome data. When not explicitly stated, outcomes were calculated based on the information included within the published record. Outcome data were presented as mean or median, and all values are expressed to 1 decimal place unless the original article did not provide this degree of accuracy. The exceptions are *p* values, which are expressed as reported in published record.

### 2.3. Outcome Measures

The primary outcome measures included complete remission (CR), partial remission (PR), progressive disease (PD), stable disease (SD), no response (NR), overall survival (OS), and event-free survival (EFS).

## 3. Results

### 3.1. Search Results

**Figure 1:**
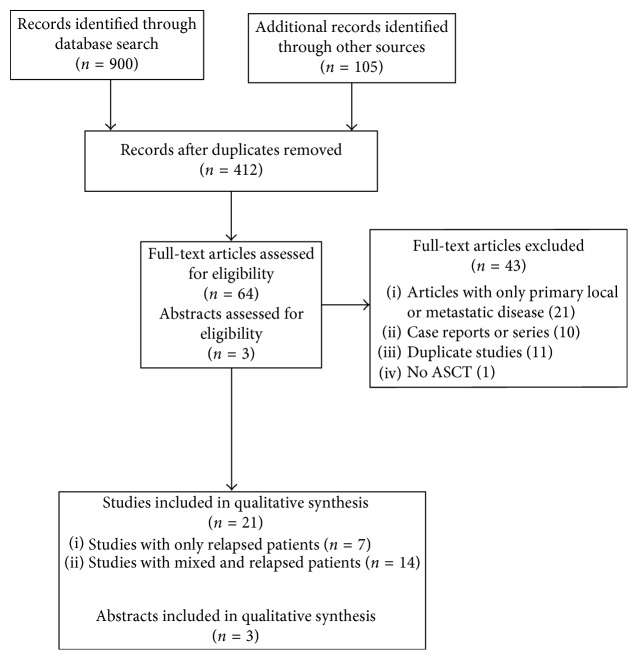


The systematic search identified a total of 1005 records. These records were screened for relevance based on their titles and abstracts. Of these studies, 64 were deemed potentially eligible and retrieved for full text review. After detailed review, a total of 43 studies were further excluded for the following reasons: duplicate study data, not focused on relapsed sarcoma, or not receiving ASCT. A total of 21 articles met our inclusion criteria. In addition, 3 published abstracts were also included (Figure 1).

### 3.2. Study Population

All studies included older children and younger adults with pathologically proven relapsed Ewing's sarcoma. Seven retrospective studies had patients with only relapsed Ewing's sarcoma that received HDCT (*n*=205) (Table 1). Fourteen studies had a mixed relapsed Ewing's sarcoma, primary metastatic disease, and other soft tissue sarcomas. In these studies, a total of at least 140 patients with relapsed ES received HDCT/ASCT. Ten of these studies reported outcomes of patients with relapsed ES separately (Table 2). Four studies reported only cumulative outcome of all patients included (Table 3). In addition, three retrospective study abstracts consisting of patients with relapsed Ewing's sarcoma and primary metastatic disease were included (Table 4).

### 3.3. Retrospective Studies Containing Only Relapsed Patient Data

We identified a total of seven retrospective studies that had only relapsed ES patients who received HDCT/ASCT (*n*=205). McTiernan et al. [15] studied 33 relapsed patients with local and metastatic disease (Table 1). All patients received CC (showed CR/PR/SD/minor response after CC) followed by HDCT/ASCT. The two- and five-year EFS rates were 43% and 39%, respectively. The OS rates were 51% and 43% at two and five years, respectively. In a study performed by Ferrari et al. [17], 20 patients that showed a disease response with CC (CR/PR) were given HDCT/ASCT, and the five-year OS rate was 50% (Table 1). Similar superior results of HDCT/ASCT (given if there was disease response with CC) following CC (*n*=24) in comparison to just CC (*n*=31) was noted by Palmerini et al. [18]. The OS at 3 years was 33% in the former group and 22% in the later group (Table 1). The median overall survival of 49 months and median relapse-free survival of 16 months was noted in another study by Shankar et al. [19] for some patients (*n*=7) with relapsed ES that showed response (CR/PR) with CC and subsequently received HDCT/ASCT (Table 1). Bacci et al. [3] studied 195 patients with relapsed ES, of which 35 patients received HDCT. The outcomes were reported for 33 patients in this study. The EFS at 5 years was 21.2%. Among these 33 patients, thirteen of eighteen patients who had surgery/radiation prior to HDCT showed response (CR/PR) before receiving HDCT. In comparison, none of the other 15 relapsed ES patients who did not receive any prior therapy (surgery/radiation/salvage chemotherapy) showed response even after getting HDCT/ASCT. This is suggestive that HDCT might not be very effective in disease that is not responsive to first-line salvage treatment at relapse (Table 1).

Barker et al. [16] studied 55 patients with local and metastatic relapse (Table 1). All patients were treated with CC, and 27 of them showed chemosensitivity (either PR/CR). Thirteen of these twenty-seven patients subsequently went on to receive HDCT followed by ASCT. The five-year EFS and OS rates for this group was 61% and 77%, respectively, whereas it was 21% and 22% for patients (*n*=14) that showed response to CC but did not receive HDCT (*p*=0.018 for both EFS and OS). Rasper et al. [14] studied 239 patients from CESS registry (cooperative Ewing's sarcoma study group) with local and metastatic relapse (Table 1). All patients received CC. In addition, 73 patients received HDCT/ASCT after CC. The outcomes were reported for 53 patients that were treated with either busulfan/melphalan or treosulfan/melphalan regimen. The outcomes were not reported for patients that were treated with consortium of other regimens (*n*=20). The two- and five-year EFS for patients who were treated with HDCT were 44–47% and 20–24%, respectively, depending on the regimen of HDCT that was used. The corresponding EFS at 2 and 5 years for patients that got only CC was 10% and 6% (*p*=0.01). The two- and five-year OS rates for patients who got HDCT were 53–66% and 40–42%, respectively, compared to 22% and 10% in patients with only CC (*p*=0.01).

To summarize, for most studies consisting of only relapsed patients that received HDCT/ASCT after CC, EFS rates at two- and five-year ranged 42–47% and 20–61%, respectively. The OS rates at two years and at three to five years ranged 50–66% and 33–77%, respectively, depending on the study. Patients in Bacci et al. [3] that did not receive salvage therapy prior to HDCT did not show any clinical response (CR/PR).

### 3.4. Studies Containing Mixture of Relapsed and Primary Metastatic Ewing's Sarcoma Patients' Data (Results for Relapsed ES Reported Separately)

There were 9 studies (8 retrospective and 1 prospective), in which 105 relapsed ES patients received HDCT/ASCT after CC. In most studies, only patients that showed response (CR/PR) to CC proceeded to get HDCT. The response status after CC was not clearly outlined in 2 studies [27, 28]. Frohlich et al. [27] studied 131 patients who were included in a registry that had 52 patients with relapsed disease. All patients received CC followed by additional HDCT/ASCT. For ES patients that relapsed within 2 years of initial disease presentation, the EFS at four years for patients that got HDCT was 17%. The study compared outcomes to historical control patients where EFS at four years was 2% with only CC (*p*=0.0001) (Table 2). Burdach et al. [22] studied 17 patients, of which 10 had relapsed ES. All patients received CC followed by HDCT/ASCT. The EFS and OS for relapsed patients at 4 years were 50% each (Table 2). Seo et al. [25] conducted a similar study on 9 patients, 5 of which had relapsed disease. At two years, EFS for relapsed patients was 40%. The OS at 1 and 2 years was 60% and 40%, respectively, for relapsed patients (Table 2). The OS was 70% reported at three years by Juergens et al. [28] in their study of 11 relapsed patients (Table 2). Similarly, Rosenthal et al. [26] studied 20 patients, 14 of whom had relapsed disease. All relapsed patients received HDCT after CC and showed EFS/OS at 1 year to be 36%/50%.

Ekert et al. [20] and Parentesis et al. [23] reported results on smaller group of relapsed patients. The OS at 2 years for the 2 relapsed patients in the former study was 50% (CC followed by HDCT), and it was 66% at three years for 3 relapsed patients who were in CR (after receiving CC) prior to HDCT/ASCT in the later study. Jahnukainen et al. [24] noted that the only relapsed ES patient in their study who received thiotepa-based HDCT following CC survived for 8.5 years. In the same study, the cumulative OS for 3 patients with relapsed Ewing's family of tumors (primitive neuroectodermal tumors and Ewing's sarcoma) at 2 years was 100% (Table 2). Graham-Pole et al. [21] studied 8 patients, 6 of which had relapsed disease. In this study, HDCT was given as induction regimen. None of the relapsed ES patients survived beyond 3 months. This study was performed in 1984 when transplant-related mortality was high, and patients received HDCT as an induction regimen without prior CC; these factors would have contributed to poor outcomes (Table 2).

### 3.5. Studies Containing Mixture of Relapsed and Primary Metastatic Ewing's Sarcoma Patients' Data (Results for Relapsed ES Not Reported Separately)

Five studies (2 prospective and 3 retrospective) reported combined outcomes of all patients in their study. At least 35 relapsed ES patients received HDCT. In most studies, patients who proceeded to get HDCT/ASCT showed initial response (CR/PR) with CC. The response status of patients was not clearly outlined in one study [31]. Al-Faris et al. [29] studied 45 patients, out of which 19 had relapsed disease. All patients received CC. Twenty patients received additional HDCT/ASCT (this included 6 patients with relapsed ES). The one-and-half-year EFS and three-year EFS for patients who received HDCT were 60 and 39%, respectively, whereas for patients who got only CC, EFS was 36 and 32% (*p*=0.08), respectively. The one and half and three-year OS for patients who received HDCT was 70% and 59%, respectively, whereas it was 44 and 34% for patients who got only CC (*p*=0.06) (Table 3). Pape et al. [31] presented results of 39 patients that were mix of primary metastatic and relapsed ES. All patients received CC followed by HDCT/ASCT. OS at three and half years was 31 percent for the whole group (Table 3). In another study by Fraser et al. [30] that consisted of sixteen patients, six had relapsed disease. Patients got CC followed by HDCT and ASCT. For patients with ESW (ES and desmoplastic small round cell tumors) at one and three years, OS was 69 and 54 percent, respectively (Table 3). Ladenstein et al. [33] performed a registry study consisting of 31 patients with relapsed EFT (Ewing sarcoma family of tumors) consisting of patients with Ewing's sarcoma and primitive neuroectodermal tumors. Twenty-four of these had relapsed ES. All patients received CC followed by HDCT and ASCT. EFS for all relapsed patients at five years was 32%. In addition, the cumulative outcome for all patients (*n*=63) included in the study (primary metastatic EFT and relapsed EFT) at 5 years was 27% (Table 3). Kavan et al. [32] reported one and half year OS to be 58% for the 31 patients (mix of advanced primary ES/relapsed ES) studied who received CC followed by HDCT and ASCT (Table 3).

To summarize, for most studies that only reported combined outcomes for patients with relapsed/primary metastatic ES along with other malignancies (that got HDCT/ASCT following CC), EFS at 1.5–3 and 5 years was 39–60% and 27%, respectively. OS at 1–1.5 and 3–3.5 years was 58–70% and 31–59%, respectively. In studies that reported outcomes for relapsed ES patients separately (that received HDCT/ASCT after CC), EFS at 1–3 and 4 years was 36–66% and 17–50%, respectively. The OS at 1–2 and 3–4 years was 40–60% and 50–70%, respectively, depending on the study. Patients in Graham-Pole et al. [21] did exceptionally poor possibly because of lack of usage of CC prior to HDCT. The three patients with relapsed EFT (including one patient with relapsed ES) along with patients with advanced primary ES in Jahnukainen et al. [24] did exceptionally well on thiotepa HDCT regimen, the reasons not being very clear.

### 3.6. Retrospective Studies (Abstracts) Containing Mixture of Relapsed and Primary Metastatic Ewing's Sarcoma Patients' Data

We also found three abstracts (*n*=42) that were published by Thiel et al. [36], Cristofani et al. [35], and Elhasid et al. [34]. All studies had a mixed group of patients consisting relapsed and primary metastatic disease. The results were also reported for combined group and not separately for relapsed patients. To summarize, the OS at three to six years ranged from 33–62% (Table 4).

### 3.7. Comparative Studies of Relapsed Ewing's Sarcoma

Five studies compared results of relapsed patients who received HDCT along with ASCT after CC to those who received only CC [3, 14, 16–18]The EFS at 2 and 5 years for patients that got HDCT/ASCT after CC was 44–47% and 20–61%, whereas it was 10% and 0–7%, respectively, for patients who got only CC. The OS at 2–5 years was 33–77% for HDCT/ASCT (after CC), whereas it was 5–22% for only CC. In addition, Bacci et al. [3] reported mOS with HDCT/ASCT (after surgery/radiation) to be 23 months compared to 11.1 months with only CC. Two studies demonstrated superior results of HDCT/ASCT in chemosensitive relapsed ES (after CC) through direct comparison of survival outcomes of patients that got HDCT (after CC) to those that did not receive it [14, 16]. They reported EFS and OS after HDCT at 2–5 years to be 22–61% and 41–77%. The EFS and OS with just CC (without additional HDCT) at 2–5 years were 18–31% and 22–45%, respectively [14, 16]. These comparative studies clearly show that HDCT/ASCT given as consolidative measure after initial salvage regimen clearly has a survival benefit (Table 5).

## 4. Discussion

In patients with relapsed Ewing's sarcoma, there is not one established regimen as the standard of care. Regarding conventional therapy salvage options, many regimens have been tried with variable results. A phase II study with ifosfamide with mesna along with etoposide showed that sixteen of seventeen patients with relapsed Ewing's sarcoma showed responses (either CR/PR). The patients in this study were only followed for 10 weeks; hence, long-term survival is not known [37]. Another phase II study using ICE regimen (ifosfamide, carboplatin, and etoposide) showed response rate of 51% and 1 -year and 2 -year OS rates of 49% and 28%, respectively [7]. Among nonifosfamide regimens, docetaxel along with gemcitabine showed overall response rate (ORR) of 29% and median duration of response of 4.8 months [6]. Cyclophosphamide and topotecan showed response rate of 44% and 2 -year EFS rate of 26% in study with 54 relapsed Ewing's sarcoma patients [8]. Irinotecan and temozolomide resulted in response rate of 63% and time to progression of eight months [10]. A study with patients receiving VIT regimen (vincristine, irinotecan, and temozolomide) showed ORR of 68% with 22.7% patients alive and with no evidence of disease at 10.3 months [9]. In another retrospective study conducted on 107 patients with relapsed Ewing's sarcoma with either etoposide and cisplatin or etoposide and carboplatin showed EFS of 6.5 and 14 months, respectively, with 5 -year OS rates of 20% and 24.5%, respectively [11]. Collectively, these data suggest the need for further improvement in treatment outcomes in Ewing's sarcoma patients.

The usage of HDCT followed by ASCT rescue is based on observation that outcome in many malignancies that are chemosensitive is dependent on the dosage of chemotherapy used. Steep dose-response is noticed for both toxic and therapeutic effects. Preclinical studies documented linear log correlation between dose and tumor cytotoxicity. An increase in dose by three to tenfold, particularly for alkylating agents, can result in a multiple log increase in tumor cell death. Myelosuppression is one of the major side effects at this dose [38]. ASCT helps to rescue marrow and allows for further dose escalation [39]. HDCT along with ASCT has shown to benefit in the treatment of various malignancies including high-grade germ cell tumors [40, 41], newly diagnosed multiple myeloma [42, 43], and relapsed Hodgkin's and non-Hodgkin lymphomas [44]. HDCT with ASCT has also shown benefits in improving survival in chemosensitive soft tissue sarcomas including relapsed osteosarcoma patients [45, 46]. HDCT/ASCT has shown benefit in improving OS and EFS for relapsed Ewing's sarcoma in single institute studies [15, 16, 22].

We compared outcomes of HDCT with ASCT given as consolidation regimen after CC to results from historical studies which used only CC. In most studies, patients with relapsed ES that received HDCT/ASCT showed initial chemosensitivity (CR/PR) to CC. The OS was 20–60% at 1-2 years and 20–25% at 5 years when only CC was used in historical studies [7, 8, 11]. The EFS at one to two years was around 25% [8, 9]. For most studies utilizing HDCT/ASCT that presented outcomes for only relapsed patients, the EFS at two and five years was 42–47% and 20–61%, respectively. The OS for this group at 2 and 3–5 years ranged from 50 to 66% and 33–77%, respectively. For studies utilizing HDCT/ASCT that consisted data on mixed patients (relapsed, primary metastatic disease, and other soft tissue sarcomas) which reported outcomes for relapsed ES separately, the cumulative EFS at 1–3 and 4 years was 36–66% and 17–50%, respectively. The OS at 1-2 and 3-4 years was 40–60% and 50–70%. The other studies consisting of mixed patients reported outcomes for the whole group. The EFS at 1.5–3 and 5 years was 39–60% and 27%, respectively. The OS from the same group at 1–1.5 and 3–3.5 years was 58–70% and 31–59%, respectively. For data presented in the abstract format, the cumulative OS (not separated for relapsed ES) at three to six years was 30–60% [34–36, 47]. As evident, the OS and EFS with HDCT followed by ASCT given after CC was better than the outcomes from historical studies where only CC was used.

Five studies that compared outcomes of patients with relapsed ES who received HDCT and ASCT after CC with those who received only CC showed improved survival with HDCT/ASCT [3, 14, 16–18]. Patients in two studies though did not show improved outcomes with HDCT. Patients in the work of Pole et al. [21] did not receive CC prior to HDCT, which might have contributed to poor outcome. This is also the possible contributing cause for poor outcomes for a subgroup of 15 patients (did not get surgery/radiation prior to HDCT) that did not show response (CR/PR) even after getting HDCT/ASCT in a study done by Bacci et al. [3] Results of both these studies suggest that HDCT probably is not very effective in disease that is not responsive to initial salvage therapy (chemotherapy or other treatments) at relapse.

Among all positive OS survival outcomes, HDCT regimens used in eight studies (Barker et al. [16], Ferrari et al. [48], Juergens et al. [28], Burdach et al. [22], Elhasid et al. [34], Al-Faris et al. [29], Jahnukainen et al. [24], and Parenthesis et al. [23]), seemed to have resulted in best OS among others. The HDCT regimen used in these seven studies are listed in Tables 1–3. Most regimens contained melphalan in combination with other cytotoxic agents. Given the heterogeneity among studies which used HDCT, it is difficult to determine if there was a superior regimen with better outcomes when compared to others. With realization of limited data, all three patients with relapsed EFT in Jahnukainen et al. [24] study received thiotepa-based HDCT and did exceptionally well and were surviving at the end of two and half years.

The superior outcomes seen in the studies could also have been impacted by many factors, such as selection bias in favor of younger, fit patients who were offered HDCT. Most studies included in our analysis were retrospective in nature, and patients who did not respond to CC did not proceed to receive HDCT, thus adding a further selection bias for chemosensitive patients, who are more likely to be offered HDCT/ASCT, which may affect the positive impact observed with this intervention. Limitation of our review also includes significant heterogeneity in included study designs, type of CC and HDCT used, local versus metastatic relapse, different inclusion criteria, follow-up duration, and study end points in various trials included in our analysis. These factors made it difficult to perform meta-analysis of the available data. In addition, patients included in studies had combined data on relapsed and primary metastatic disease. Despite these limitations, results of this review suggest that HDCT has a positive impact on survival.

## 5. Conclusion

In conclusion, HDCT and ASCT given as a consolidation treatment after CC appear to be beneficial in improving survival in patients with relapsed Ewing's sarcoma. It appears to be more prominent in chemosensitive diseases. HDCT given as induction treatment did not appear to be beneficial in improving survival, but it was used in only two studies. The heterogeneity in the included studies suggests that prospective randomized controlled studies are needed to definitively show the benefit for single and two cycles of HDCT and ASCT in relapsed Ewing's sarcoma.

## Figures and Tables

**Table 1:** Studies consisting of patients with only relapsed Ewing's sarcoma.

Study	Median age (years)	Number of pts	Type of disease (number of pts)	Type of relapse (number of pts)	Chemotherapy	HDCT (mg/m^2^; mg/kg)	Number of HDCT pts	Source of stem cell	Response (number of pts)	EFS	OS
Rasper et al. 2014, registry study [14]	NL	239	Local (42) Metastatic (197)	Local (42) Distant (142) Combined (30)	T/C I/Te If/E/carboplatin	C1: busulfan/melphalan (NL) C2: treosulfan/melphalan (NL)	73 C1 = 15 C2 = 38 COR = 20 (ONR)	NL	Prior to HDCT: C1: CR (5) PR (2) C2: CR (19) PR (8)	2 yr HDCT: 44–47% 5 yr HDCT: 20–24%	2 yr HDCT: 53–66% 5 yr HDCT: 40–42%

McTiernan et al. 2006, retrospective [15]	19	33	Local (22) Metastatic (11)	Local (11) Distant (18) Combined (4)	Ca E C A I D V	Busulfan/melphalan (600 mg/m^2^/140 mg/m^2^) (22 pts) Melphalan/etoposide (130 mg/m^2^/60 mg/m^2^) (7 pts) TBI Melphalan/cytoxan (16 mg/kg/60 mg/kg) (1 pts) Melphalan (150 mg/m^2^) (3 pts)	33	PBSC BSC	Prior to HDCT: CR (14) PR (10) Post HDCT: CR (21) PR (2)	2 yr EFS 42.5% 5 yr EFS 38.5% (HDCT)	2 yr OS 50.7% 5 yr OS 42.8% (HDCT)

Barker et al. 2005, retrospective [16]	13.5	55	Local (30) Metastatic (25)	Local (6) Distant (39) Combined (10)	VDC-IE VACIME VDC-A VDC-A-M	Busulfan/melphalan/THIO (NL)	13	PBSC	Prior to HDCT CR/PR (27)	5 yr HDCT: 61% (HDCT)	5 yr HDCT: 77% (HDCT)

Ferrari et al. 2015, retrospective [17]	NL	107	NL	Local (11) Distant (96)	High I VDCAIE	Busulfan/melphalan (4 mg/kg and 140 mg/m^2^)	20	PBSC	NL	NL	5 yr OS HDCT: 50%

Palmerini et al. 2009, retrospective [18]	17	72	NL	Local (11)	NL	Busulfan/melphalan (4 mg/kg and 140 mg/m^2^) Palliative	24	NL	NL	NL	3 yr. HDCT: 33%

Shankar et al. 2003, retrospective [19]	14	64	Local (64)	Local (11)	Ca/E C/E/Ca C/E	Melphalan (NL) TBI	7	PBSC BSC	NL	mRFS = 16 months	mOS = 49 months (HDCT)

Bacci et al. 2003, retrospective [3]	18	195	NL	Local (57) Distant (138)	None	Melphalan (NL) Busulfan (NL)	35 ONR = 2	PBSC BSC	NL	5 yr with HDCT: 21.2%	mOS with HDCT: 27.1 m

TBI: total body irradiation, HDCT: high-dose chemotherapy, SCT: stem cell transplant, CR: complete response, PR: partial response, EFS: event-free survival, OS: overall survival, V: vincristine, E: etoposide, M: methotrexate, A: dactinomycin, If: ifosfamide, C: cyclophosphamide, D: doxorubicin, T: topotecan, I: irinotecan, Te: temozolomide, Ca: carboplatin, NL: not listed, high I: high-dose ifosfamide, mOS: median overall survival, mRFS: median relapse-free survival, COR: consortium of other regimens, and ONR: outcomes not reported.

**Table 2:** Studies consisting of patients with mixture of relapsed and primary metastatic Ewing's sarcoma (outcomes of patients with relapsed Ewing's sarcoma reported separately).

Study	Average age (years)	Total number of pts	ES number pts	Number of HDCT patients (ES rel)	Chemotherapy	Response (number of pts)	HDCT (mg/m^2^; mg/kg)	Source of stem cell	EFS/RFS	OS
Ekert et al. 1984, retrospective [20]	12	22	Total (4) Relapsed (2)	2	V A Dacarbazine	CR (1)	V (1.5 mg/m^2^) Cytoxan (1200 mg/m^2^) Imidazole (250 mg/m^2^) A (20 mg/m^2^)	BSC	Rel-1 yr RFS-0%	Rel-2 yr OS 50% (HDCT)

Pole et al. 1984, retrospective [21]	15	18	Total (8) Relapsed (6)	5	V Cytoxan A Actinomycin	RP.SA	Melphalan (120–210 mg/m^2^)	BSC	Rel-mEFS (HDCT)-3 months	NL

Burdach et al. 1993, retrospective [22]	16	17	Total (17) Relapsed (10)	10	Cytoxan I VP16	CR (6) PR (4)	Melphalan (120–180 mg/m^2^/dose) Etoposide (60 mg/kg) TBI	PBSC BSC	Rel-4 yr EFS 50% All pts: 6 yr-45% (HDCT)	Rel-4 yr OS 50%

Parentesis et al. 1999, retrospective [23]	5–28	24	Total (9) Relapsed (5)	5 (3 pts. in CR)	None	CR (3) PR (2)	Melphalan (50 mg/m^2^) Thiotepa (300 mg/m^2^) Busulfan (1 mg/kg) Etoposide (1800 mg/m^2^)	PBSC BSC	Rel-3 yr. EFS 66 % (CR) (HDCT)	Rel-3 yr OS 66% (CR)(HDCT)

Jahnukainen et al. 2015, retrospective [24]	NR	24	Total (9) Relapsed ES (1) Relapsed EFT (3)	1	Cytoxan D DactinomycinI	CR (1)	Thiotepa (900 mg/m^2^)	BSC	NL	Rel (EFT, *N*=3) 2 yr OS = 100% Rel (ES, *N*=1)-8.5 years (HDCT)

Seo et al. 2013, retrospective [25]	13.4	9	Total (9) Relapsed (5)	5	V I D	CR (4) SD (1)	Cytoxan (1500 mg/m^2^/day) Melphalan (60 mg/m^2^/day)	PBSC	Rel-2 yr EFS 40% All pts: 2 yr EFS 45% (HDCT)	Rel-1 yr OS 60%/2 yr OS 40% All pts: 2 yr OS 45% (HDCT)

Rosenthal et al. 2008, prospective single arm [26]	16	20	Total (20) Relapsed (14)	14	V A ICE	CR (9) PR (2) SD (1) PD (2)	C1: melphalan/busulfan C2: melphalan/carboplatin or busulfan or others Melphalan (140 mg/dose)	BSC	Rel-1 yr. EFS-36% All pts: 1 yr EFS 45%/3 yr EFS 47% (HDCT)	Rel-1 yr. OS-50% All pts: 1 yr OS 60%/3 yr OS 45% (HDCT)

Frohlich et al. 1999, retrospective [27]	NL	131	Total (131) Relapsed (52)	52	NL	NL	Melphalan (30 mg/m^2^/day, d1-4) Etoposide (1800 mg/m^2^, d1) TBI	PBSC BSC	Rel-HDCT: 4 yr EFS 17% Without HDCT: 4 yr EFS 2% (historical control patients)	NL

Juergens et al. 2009, retrospective [28]	NL	32	Total (32) Relapsed (11)	11	NL	NL	Melphalan (140 mg/m^2^) Treosulfan (36 g/m^2^)	NL	NL	Rel-3 yr OS 70% (HDCT)

HDCT: high-dose chemotherapy, TBI: total body irradiation, CR: complete response, PR: partial response, PBSC: peripheral blood stem cell, BSC: bone marrow stem cell, SCT: stem cell transplant, EFS: event-free survival, OS: overall survival, NL: not listed, M: melphalan, V: vincristine, E: etoposide, C: carboplatin, B: busulfan, P: procarbazine, I: ifosphamide, A: adriamycin, D: doxorubicin, ES: Ewing's sarcoma, rel: relapsed, RP.SA: response present, specifics unavailable, RFS: relapse-free survival, EFT: Ewing's family of tumors, and ES rel: patients with relapsed ES that got HDCT.

**Table 3:** Studies consisting of patients with mixture of relapsed and primary metastatic Ewing's sarcoma (combined outcomes for all patients reported).

Study	Average age (years)	Total number of pts	ES (number of pts)	Number of HDCT patients (ES rel)	Chemotherapy	Response (number of pts)	HDCT (mg/m^2^; mg/kg)	Source of stem cell	EFS	OS
Al-Faris et al. 2007, retrospective [29]	12.5	45	Total (45) Relapsed (19)	6	TC ICE V D	RP.SA	Etoposide (2400 mg/m^2^) Cytoxan (60 mg/kg/dose) Melphalan (180 mg/m^2^/dose)	PBSC	All pts: HDCT: 1.5 yr EFS 60%/3 yr EFS 39% Without HDCT: 1.5 yr EFS 36%/3 yr EFS 32%	All pts: HDCT: 1.5 yr OS 70%/3 yr OS 59% Without HDCT: 1.5 yr OS 44%/3 yr OS 34%

Fraser et al. 2006, prospective single arm [30]	11.5	36	Total (16) Relapse (6)	5	ICE V C	CR (3) PR (3)	Melphalan (50 mg/m^2^) Busulfan (4 mg/kg) Thiotepa (250/m^2^)	PBSC BSC	NL	ES family-1 yr OS 69%/3 yr. OS 54% (HDCT)

Pape et al. 1999, retrospective [31]	NL	39	39 No information on rel pts	NL	EVAIA	NL	Melphalan (30 mg/m^2^) Etoposide (1800 mg/m^2^)	PBSC	NL	All pts: 3.5 yr OS 31% (HDCT)

Kavan et al. 1999, prospective single Arm [32]	12.6	31	Total (27)	NL	NL	NL	Melphalan (NL) Etoposide (NL) Carboplatin (NL) TBI	NL	NL	All pts: 1.5 yr OS 58% (HDCT)

Ladenstein et al. 1995, registry [33]	13	63	Total (50) Relapsed (24) Relapsed EFT (31)	24	VAC/VAD IVA/IVAD	CR (24)	G1: M + V or E + C (NL) G2: M + B ± Cy(NL) G3: (NL) M + Ba + V M + Ba + P M + Ba + T G4 (NL) Th + V + I + Ci G5 (NL) M + V + TBI M + C + TBI M + Ci + TBI	PBSC BSC	All pts: 5 yr-27% Relapsed pts (EFT) (*N*=31)-5 yr EFS 32% Primary MET pts-5 yr EFS-21% (HDCT)	NL

TC: topotecan and cyclophosphamide, ICE: ifosfamide, carboplatin, and etoposide, EVAIA: etoposide, vincristine, adriamycin, ifosfamide, and actinomycin D, HDCT: high-dose chemotherapy, TBI: total body irradiation, CR: complete response, PR: partial response, PBSC: peripheral blood stem cell, BSC: bone marrow stem cell, EFS: event-free survival, OS: overall survival, NL: not listed, V: vincristine, E: etoposide, C: carboplatin, T: teneposide, I: ifosphamide, A: adriamycin, D: doxorubicin, M: melphalan, V: vincristine, B: busulfan, Cy: cyclophosphamide, Ba: bichloronitrosourea, P: procarbazine, Ci: cisplatin, A: adriamycin, ES: Ewing's sarcoma, rel: relapsed, RP.SA: response present, specifics unavailable, RFS: relapse-free survival, and ES rel: patients with relapsed ES that got HDCT.

**Table 4:** Retrospective abstracts consisting of mixture of patients with primary metastatic and relapsed Ewing's sarcoma.

Study	Average age (years)	Number of pts	Chemotherapy	HDCT	Number of Tx prior to SCT	Source of stem cell	Response (number of pts)	EFS	OS
Elhasid et al. 2012, retrospective [34]	14	Total (16)	NL	Melphalan (NL) Busulfan (NL)	NL	NL	NL	8 yr EFS 41%	6 yr OS 56%

Cristofani et al. 2013, retrospective [35]	7	Total (18)	NL	NL	NL	PSBC BSC	NL	NL	5 yr OS 33%

Thiel et al. 2014, retrospective [36]	NL	Total (8) Relapsed (2)	NL	NL	NL	NL	CR (7)	Median EFS 16.5 months	2.5 yr OS 62%

NL: not listed, HDCT: high-dose chemotherapy, SCT: stem cell transplant, PBSC: peripheral blood stem cell, BSC: bone marrow stem cell, EFS: event-free survival, OS: overall survival, and CR: complete remission.

**Table 5:** Studies comparing outcomes of HDCT and CC.

Study	Number of relapsed pts (only CC)	Number of relapsed pts (HDCT)	Chemotherapy (prior to HDCT)	HDCT	Source of stem cell	Response CC (number of patients)	Response HDCT (number of patients)	EFS	OS
Rasper et al. 2014, registry data [14]	161	53	T/C I/Te If/E/Ca	C1: busulfan/melphalan C2: treosulfan/melphalan	NR	CR: 14 PR: 20 SD: 12 PD: 72 MD: 43	C1 CR: 5 PR: 2 C2 CR: 19 PR: 8 (before HDCT)	2 years HDCT: 44–47% HDCT IN CR/PR: 44% Without HDCT: 10% Without HDCT IN CR/PR: 31% 5 years HDCT: 20–24% HDCT IN CR/PR: 22% Without HDCT: 6% Without HDCT IN CR/PR: 18%	2 years HDCT: 53–66% HDCT IN CR/PR: 59% Without HDCT: 22% Without HDCT IN CR/PR: 45% 5 years HDCT: 40–42% HDCT IN CR/PR: 41% Without HDCT: 10% Without HDCT IN CR/PR: 25%

Barker et al. 2005, retrospective [16]	42	13	VDC-IE VACIME VDC-A VDC-A-M	Busulfan/melphalan/thiotepa	PBSC	CR/PR: 14	CR/PR: 13 (before HDCT)	5 years HDCT: 61% Without HDCT: 7% (*p* < 0.0001) Without HDCT IN CR/PR: 21% (*p*=0.018)	5 years HDCT: 77% Without HDCT: 7% (*p* < 0.0001) Without HDCT IN CR/PR: 22% (*p*=0.018)

Palmerini et al. 2009, retrospective [18]	31	24	NL	Busulfan/melphalan	NL	NL	NL	NL	3 yr. HDCT: 33% Without HDCT: 22%

Ferrari et al. 2015, retrospective [17]	60	20	High I	Busulfan/melphalan	PBSC	CR: 9 MD: 4	CR/PR: 20 (before HDCT)	NL	5 yr. HDCT: 50% Without HDCT: 5–12%

Bacci et al. 2003, retrospective [3]	98	35 RO: 33	None	Melphalan Busulfan	PBSC BSC	CR/PR: 2%	CR/PR-39% (after HDCT)	5-year HDCT–21.2% Without HDCT: 0%	mOS HDCT–27.1 m Without HDCT 11.1 m

HDCT: high-dose chemotherapy, CC: conventional chemotherapy, ASCT: autologous stem cell transplant, OS: overall survival, EFS: event-free survival, CR: complete remission, PR: partial remission, NL: not listed, T: topotecan, C: cyclophosphamide, I: irinotecan, Te: temozolomide, V: vincristine, E: etoposide, M: methotrexate, A: dactinomycin, If: ifosfamide, D: doxorubicin, Ca: carboplatin, TBI: total body irradiation, high I: high-dose ifosfamide, SD: sustained disease, PD: progressive disease, MD: missing data, and RO: reported outcomes.
